# Risk of very serious injuries in Swedish scaffolders - What is an acceptable risk?

**DOI:** 10.1186/s40621-026-00717-x

**Published:** 2026-09-14

**Authors:** Bengt Järvholm, Hans Pettersson

**Affiliations:** Department of Epidemiology and Global Health, Umeå University, Umeå, SE-901 87 Sweden

**Keywords:** Fall from height, Construction industry, Subcontractors

## Abstract

**Background:**

This case study estimates the risk of injuries causing death or at least one year’s sick leave due to fall from height among Swedish scaffolders.

**Findings:**

Cases occurring in 2020 to 2023 were identified from national statistics of accidents at work and statistics from an insurance company. The number of scaffolders in Sweden were estimated through national statistics. Their risk was compared to what is considered an unacceptable risk for exposure to carcinogens. Between 2020 and 2023 there were 10 accidents due to fall from height causing death or at least one years’ sick leave among Swedish male scaffold workers. The average number of scaffolders at risk were estimated to around 3,100 per year indicating a risk of at least 3% for such an injury for a person working as a scaffolder from 20 to 65 years of age.

**Conclusions:**

The estimated risk of very serious injuries in Swedish scaffolders is today around ten times higher than a prohibition risk level for exposure to direct-acting carcinogens according to a Dutch document used for setting maximum allowable concentrations due to occupationalr exposure.

## Introduction

Falls from height are common causes for serious injuries in the construction industry [1]. Scaffolds are used on most construction sites and are usually built and demolished by special workers, scaffolders. They have mostly special training. In Sweden, they must undergo training or receive special information on scaffolding to get a license. Their skills include knowledge about working on temporary worksites, at height, using safety equipment and how to make a safe scaffold. There are several studies suggesting methods to make their work safer often through “safe behaviour” [2, 3]. For work environment risk management, it is crucial to understand what the risks are for serious injuries and therefore prioritize to manage these risks at work.

The current study is part of a larger study which has the purpose to demonstrate the use of injury data from registers in the routine work of the Swedish Work Environment Authority. The project used recent data from two registers and focused partly on the construction industry. The results described in this case report were not planned but became obvious during the analysis of the data as several cases of serious injuries in scaffolders were noticed. There is no data on what the risk is of severe injury among scaffolders in Sweden nor how many current scaffolders have severe injuries due to fall. This paper describes the findings, estimate the risk and compare it with a classification of acceptable risks in occupational settings.

## Methods

An occupational injury in Sweden can be compensated through the general social insurance (SI). It compensates loss of income in all workers who pay taxes in Sweden. Fatal occupational injuries reported to SI are the basis for the register of fatal occupational injuries organized by the Swedish Work Environment Authority (SWEA). Information of the causes to injuries are mostly provided by the employer [4]. However, its register includes no data on period of sick leave due to occupational injuries.

There is also a collective agreed insurance covering about 90% of all employed workers. It provides additional compensation for occupational injuries. They can be compensated from the first day and in addition to financial losses, it compensates for pain, loss of glasses etc. Its register includes data on total sick leave due to an injury. The register contains a case with sick leave data only when the sick leave due to the injury had come to an end. The analysis in this report uses information from injuries in the register occurring between 2020 and 2023. Data from the register was transferred for this project in spring 2025. The major cause of an injury is classified by the register and mostly based on information from the injured worker. This insurance is through an insurance company owned by social partners (Afa Insurance [5]). The focus in this analysis was on injuries classified as caused by fall from height among scaffolders. In the Afa Insurance register scaffolders have a unique code (7116).

Information about the size of the employed work force that are scaffolders, during the study period, were gathered from the Swedish occupational register in Statistics Sweden [6].

The study was approved by the Swedish Ethical Review Authority (DNR 2024–05595).

## Results

### Cases

There were 269 injuries among scaffolders in the Afa register 2020–2023 of which all but one were among men. Of these 269 cases 44 were classified as fall from height, all among men. Seven of the 44 cases had sick leaves of more than a year. Their median age were 36 years, five were younger than 40 years of age and two were between 50 and 59 years of age.

There were in 2020–2023 three fatal injuries in scaffolders in the national register of occupational fatalities of SWEA [4]. They occurred in 2020 (one case) and 2022 (two cases). All were described as a falls from height and all were men between 29 and 35 years.

According to the register at Statistics Sweden there were between 3258 and 3861 men and 24–69 women that were scaffolders in 2020 to 2024, Table A1 (appendix).

### Risk estimation

If a fatal injury or an injury causing at least one year of sick leave is classified as “very serious”, there were 10 very serious injuries in 2020 to 2023, i.e. 2.5 very serious injuries per year.

The population at risk is somewhat more uncertain. A fatal injury among a scaffolder would with high probability be included in the statistics of fatal injuries. The scaffolder with at least one-year sick leave due to an injury from a fall from height, would be included if his employer had a collective agreement. About 90% of the workforce is estimated to have had such an agreement. Thus, around 3,100 persons per year were estimated to be scaffolders and included in the register of the Afa insurance company if they had a very serious occupational injury. A risk of 2.5 serious injury in 3,100 persons per year would mean a risk of 0.00081 per year or a risk of around 3.1 to 3.6% during a working life of 40–45 years, assuming the same risk every year.

## Discussion

Sweden has a national zero-vision of fatal occupational injuries since 2016. The average is 40.6 cases per year (2016–2024) with no decreasing trend [4]. Any acceptable level of fatal injuries would be very hard to defend. For occupational diseases, caused by chemicals, it is rather common to regulate exposure by different measures as maximum allowable concentrations or threshold limit values. In Sweden we follow recommendations from The Dutch Health Council on chemicals. The council suggests a prohibition level for carcinogens that cause “four additional cases of cancer due to occupational exposure over a 40-year working life period per 1,000 cases from all causes”. This equates to a risk of 0.004 associated with exposure throughout a person’s working life [7]. The point estimate of lifetime risk of Swedish Scaffolders during 2020 to 2023 exceeds that level with at least ten times. This might be an underestimation as most scaffolders are rather young and high force on impact, from falling, is more lethal for elderly persons. From analysis of car crashes, the risk of similar force as from falling, is 2–3 times more often lethal in a person of age 60 years than in a person in his 20s [8]. Furthermore, a few cases with very long sick leave occurring late in the study period may have been missed if the sick leave continued after the data was sent for research, meaning that some cases with very long sick leave may have been missed (i.e. a case with an injury occurring in autumn 2023 with a sick leave of 2 years would have been missed).

However, this study is an observational study with no prior hypotheses. Furthermore, the definition of a sick leave of at least a year was not a prior definition at the start of study.

An analysis of lethal occupational injuries reported in the national register between 2008 and 2025 lists in total 3 Swedish cases in addition to the three cases reported above [4]. There was only one other lethal injury in the register of a scaffolder due to fall from height in in the period 2008–2025 (it occurred in 2008). In addition, two scaffolders had lethal injuries due to falling objects. There are three cases reported in register (2018, 2019 and 2023) where workers from other European countries had a lethal injury where “scaffold” is mentioned. It is not clear whether they were scaffolders or had other jobs. Another 13 lethal injuries 2008–2025 mentioned “scaffold” as an object in the causation of which 12 explicitly mentioned that there was a fall from the scaffold but with other job titles. The number of workers behind these figures are unknown, i.e. the number of persons working on scaffolds on height. However, probably most construction workers are sometime on a scaffold during a year. There have yearly been around 300 000 workers in the Swedish construction industry 2008–2025, i.e. around 100 times more than the number of scaffolders. Thus, a crude estimation of the average risk for a fatal injury from a fall from a scaffold among a worker in the construction industry would be considerably lower than the scaffolders’ risk.

Thus, the occurrence of lethal injuries due to falls from height among scaffolders seem to have been higher later in the period 2008–2025, although based on small numbers. These data on lethal injuries due to fall from height were reported to the Swedish Work Environment Authority in November 2025. Discussions with persons familiar with the praxis in the maintenance of scaffolds indicate that although the use of equipment that use fixed fences during building scaffolds are recommended the use has decreased. Personal protection through safety harness is difficult at height from around 2 to 4.7 m but commonly used. There may also be organisational factors, i.e. new firms are established but may lack the necessary skills and leadership.

Injuries are signs of errors in the production and low occurrence of injuries can be regarded as efficient and skilful production. Working on heights is an obvious risk for everyone and preventive measures like railings, safety training, organization activities as licensing and personal protective equipment are common. Risk estimates can hint if the measures are sufficient and can also indicate areas for improvement. Swedish statistics are of good quality and availability. However, it must be interpreted. This study indicates that limited analysis of existing statics can contribute with knowledge of importance for prevention of serious occupational injuries.

## Data Availability

Data on cases with fatal injuries are available on www.AV.se. Information. Further data on non-fatal cases are restricted due the ethical approval.

## Appendix

**Table A1:** Number of employed scaffolders (code 7116) in Sweden according to Statistics Sweden (data downloaded 2026-02-03)

Men	2020	2021	2022	2023
	3 258	3 265	3 271	3 861
Women	24	30	35	53
